# Targeting mitochondrial damage: shining a new light on immunotherapy

**DOI:** 10.3389/fimmu.2024.1432633

**Published:** 2024-07-22

**Authors:** Wenjuan Zeng, Menghui Wang, Yuxin Zhang, Taicheng Zhou, Zhen Zong

**Affiliations:** ^1^ Department of Gastrointestinal Surgery, The 2Affiliated Hospital, Jiangxi Medical College, Nanchang University, Nanchang, Jiangxi, China; ^2^ Huan Kui Academy, Jiangxi Medical College, Nanchang University, Nanchang, Jiangxi, China; ^3^ The Second Clinical Medical College, Jiangxi Medical College, Nanchang University, Nanchang, Jiangxi, China; ^4^ Department of Gastroenterological Surgery and Hernia Center, The Sixth Affiliated Hospital, Sun Yat-sen University, Guangdong Provincial Key Laboratory of Colorectal and Pelvic Floor Diseases, Guangzhou, China

**Keywords:** mitochondrial damage, immunotherapy, tumor microenvironment, target, immune cells

## Abstract

Mitochondrial damage has a particular impact on the immune system and tumor microenvironment, which can trigger cell stress, an inflammatory response, and disrupt immune cell function, thus all of which can accelerate the progression of the tumor. Therefore, it is of essence to comprehend how the immune system function and the tumor microenvironment interact with mitochondrial dysfunction for cancer treatment. Preserving the integrity of mitochondria or regulating the function of immune cells, such as macrophages, may enhance the efficacy of cancer therapy. Future research should concentrate on the interactions among mitochondria, the immune system, and the tumor microenvironment to identify new therapeutic strategies.

## 1 Introduction

Mitochondria are the sites of aerobic respiration, which take place fundamental metabolic processes including the metabolism of proteins and nucleic acids (1). Mitochondrial damage is intimately associated with an elevated likelihood of developing a variety of diseases, including cancer and immune system dysfunction (2). The association between mitochondrial damage and the immune system as well as the tumor microenvironment (TME) has drawn an extensive amount of attention more recently. Previous researches revealed that mitochondria are essential for the normal activation, signaling, and management of the immune system (3). Mitochondrial damage can lead to mitochondrial dysfunction to trigger cellular stress and inflammatory responses, which impact the immune system (4).

TME encompasses vascular endothelial cells, immune cells, fibroblasts, non-cellular components, and more surrounding the tumor cells. It becomes an imperative target for cancer treatment and medication research because it provides essential conditions for tumor development and metastasis (5). According to recent research, many proteins that exists on mitochondria-associated membranes and can manipulate the tumor immune microenvironment. Mitochondrial damage can accelerate tumor progression through a variety of mechanisms to affect prognosis (6). Furthermore, the hypoxic, acidic TME brought on by mitochondrial damage elevates the possibility of cancer development and metastasis (7).

In summary, mitochondrial damage has considerable impacts on the TME and immune system. In this review, we first summarize the molecular mechanisms of mitochondrial damage, along with its connection with the immune system and the TME. Through investigating the interactions among mitochondria, the immune system, and the TME, our ultimate objective is to provide novel perspectives and strategies for enhancing immune regulation and cancer therapy.

## 2 The role of mitochondrial damage in changing tumor microenvironment

**Figure 1:**
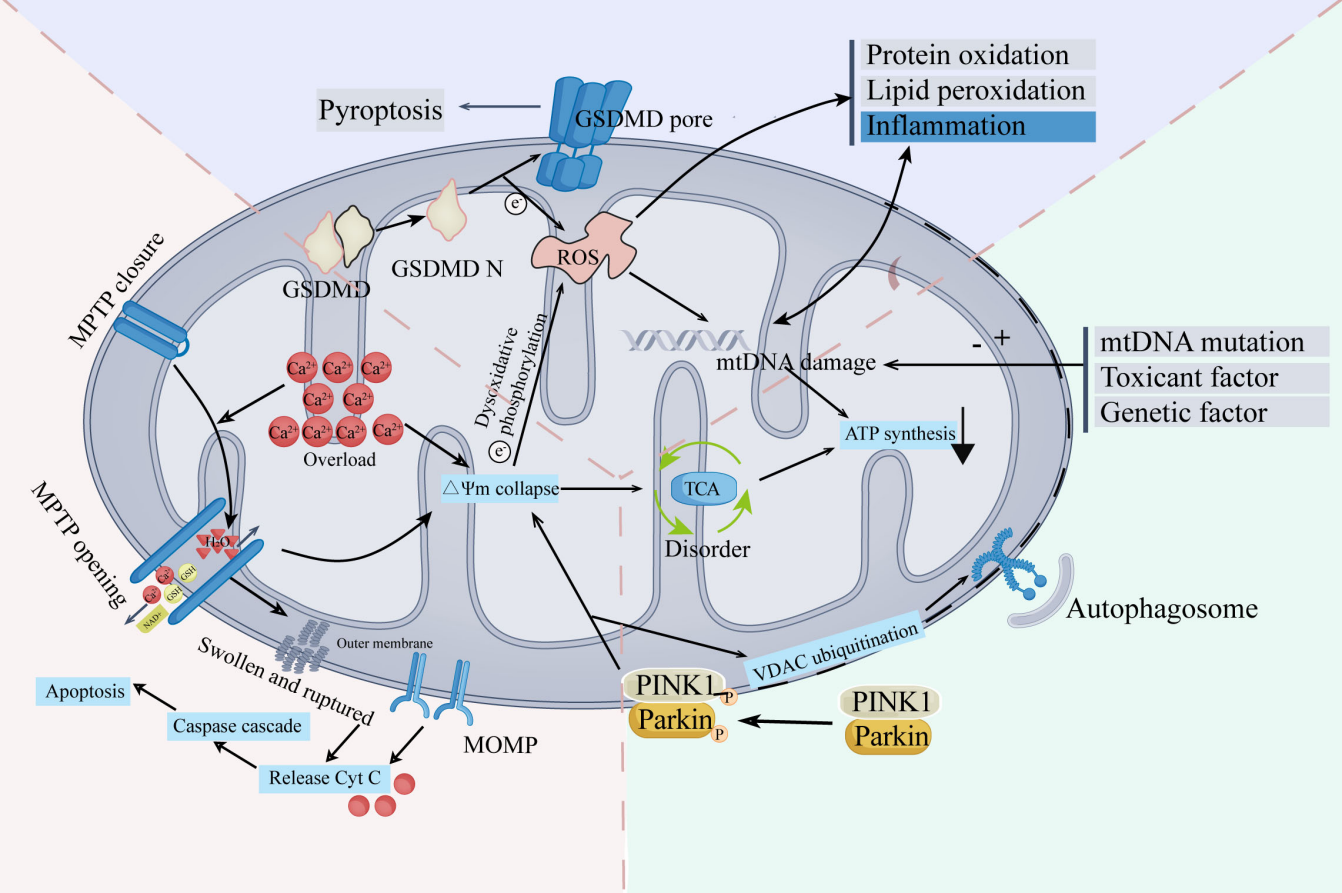
The main mechanisms and characteristics of mitochondrial damage.

The mechanism of mitochondrial damage is complex and comprehensive (**Figure 1**). Mitochondrial damage and TME interact and affect each other. A deterioration in TME stability might induce mitochondrial damage. Meanwhile, mitochondrial damage performs a crucial role in modifying TME, affecting the immune system, ferroptosis, and the conduction of calcium ions. Elevated levels of ROS, a crucial characteristic of mitochondrial damage, have a wide range of influences on the TME (8). In the following sections, we will explore the interaction between mitochondrial damage and TME from three perspectives: the ramifications of mitochondrial damage in the immune cells, ferroptosis in tumors, and the role of Ca^2+^ in changing TME.

### 2.1 The characteristics of TME and the role of mitochondria in TME

TME, a crucial environmental factor for the development and progression of cancer, is generated by the interaction of malignant and non-transformed cells in tumor tissues (9). Various cells and elements coexist in this complicated ecosystem, including immune cells, endothelial cells, and fibroblasts (10). The composition and characteristics of TME vary depending on the tumor’s location in the body, the tumor’s stage, the patient’s clinical characteristics, and other factors (10). However, the primary function of TME remains constant, coordinating tumorigenesis and malignant progression (10, 11). To support the development of tumors, satisfy the elevated metabolic requirements, and promote the growth of blood vessels, TME plays a vital role in evading immune attack and acquiring oxygen and nutrients (12, 13). Nevertheless, TME is not a balanced microenvironment. It has been recognized to be nutrient-deficient and hypoxic, acidic, exhibiting heightened oxidative stress and imbalances in electrolytes (14).

In the development of cancer and TME, mitochondria play crucial roles. Series studies indicate that mitochondrial damage may be a valuable reference to the diagnosis of cancer (15–17). Mitochondria is an essential energy factory, producing the energy for the growth and activity of cells. Additionally, mitochondria conduct signal transductions in a variety of metabolic activities, including nucleic acid synthesis, amino acid metabolism, fatty acid synthesis, and oxidation (18). The development and evolution of tumors, as well as the formation of TME, are significantly affected by the metabolic interactions among various cells (19). However, mitochondrial damage, characterized by increasing glycolysis and decreased oxidative phosphorylation (OXPHOS), may exacerbate hypoxia and the shortage of energy (20–22).

The changes in the TME may contribute to mitochondrial damage. The anoxic environment in TME emerges as a result of limited oxygen supply and elevated oxygen consumption of tumor tissues, and mitochondria are particularly sensitive to hypoxia. Depending on the oligomerization modification, protein dynamin-related protein 1 (Drp1) can bind different proteins in the ischemia and hypoxia microenvironment, which directly lead to mitochondrial damage, altering the regulation of metabolism, and causing mitophagy and mitochondrial fission (23). Furthermore, the mitochondrial damage will worsen the hypoxic environment, resulting in a vicious cycle. Additionally, studies found that the persistent inflammation in T cells will contribute to the dysfunction in mitochondrial biogenesis, partially facilitated by Akt-regulated suppression of Foxo-induced PPARgamma-coactivator-1α (PGC1α) transcription (24).

### 2.2 Mitochondrial damage in immune cells

#### 2.2.1 Effects of mitochondrial damage on macrophages

Tumor-associated macrophages (TAMs) are thought to be one of the primary constituents of TME. Studies revealed that mitochondrial damage in macrophages is crucial in the development, invasion, and metastasis of tumor tissues (25), affecting the efficacy of phagocytosis, altering the immune responses, and modifying the metabolism (26, 27).

Typically, macrophages are classified into two categories: M1 macrophages and M2 macrophages. M1 macrophages, which mostly acquire energy sources by glycolysis, are essential cells that help eradicate viruses, infections, and cancerous cells (28, 29). M2 macrophages, which obtain sources of energy primarily by oxidative phosphorylation (OXPHOS), can eliminate damaged cells while also promoting the growth of tumors (30, 31). Glycolysis plays a defensive role in macrophage activity, controlling inflammation and promoting phagocytosis, while OXPHOS is associated with lipid metabolism and the capability of anti-inflammation in macrophages (32). The transition from M1 to M2 macrophages is a significant feature of TME, causing the disorder in the immunological microenvironment and promoting the proliferation and metastasis of tumor cells (33). Besides, significantly decreased rates of glycolysis and increased OXPHOS were also observed in macrophages with mitochondrial damage (34). However, routes for polarizing macrophages vary in different types of tumor cells. Generally, mitochondrial damage will lead to an increased level of ROS (35), which can induce the production of inflammatory mediators, including IL-4, IL-6, PPARgamma-coactivator-1 beta (PGC-1β), and other factors. Macrophages will be activated by IL-4, leading to an increase in the oxidative metabolism and the activation of PGC-1β, which will stimulate the transcriptional actions of signal transducer and activator of transcription 6 (STAT 6), polarizing macrophages to M2 macrophages and reducing inflammation (36). In gastric malignancies, tumor development and macrophage M2 polarization are induced by exosome-derived circATP8A1 via the circATP8A1/miR-1-3p/STAT6 axis (37). Additionally, glioblastoma stem cells exosomal miR-374b-3p can also induce macrophages to M2 macrophages, releasing pro-angiogenic factors, including fibroblast growth factor and vascular endothelial growth factor, and leading to the angiogenesis of tumor (38), this will ultimately stimulate the growth of vascular endothelial cells and promote delivering nutrients and oxygen to the growth of tumor (39).

On the other side, the phagocytosis of macrophages also changes. Phagocytosis is a crucial function of macrophages, eliminating tumor cells and diseased cells. Damage to mitochondria will limit the activity of phagocytosis in macrophages. Studies demonstrated that to clear the apoptotic cells (ACs), macrophages require mitochondrial fission, which is mediated by Drp 1 (38). However, when mitochondria are damaged, the clearance of ACs will be hampered (40). In addition, hypoxia in TME may suppress the activation and proliferation of T cells, which substantially reduces macrophages’ ability to phagocytosis via STAT3 and its transcriptionally controlled products (41). In addition to the important role of STAT6 in macrophage polarization, STAT1 is also a key member of the STAT family, which also plays an important role in the process of macrophage polarization (42). STAT1 is mainly involved in the regulation of classically activated M1-type macrophages polarization (41). When macrophages are stimulated externally, activated STAT1 enters the nucleus and binds to the promoter region of specific genes to promote transcription, resulting in the production of many M1-type macrophage signature molecules (43). Thus, activation of STAT1 promotes polarization of M1-type macrophages, giving them pro-inflammatory and bactericidal properties.

#### 2.2.2 Effects of mitochondrial damage in T cells and B cells

**Figure 2:**
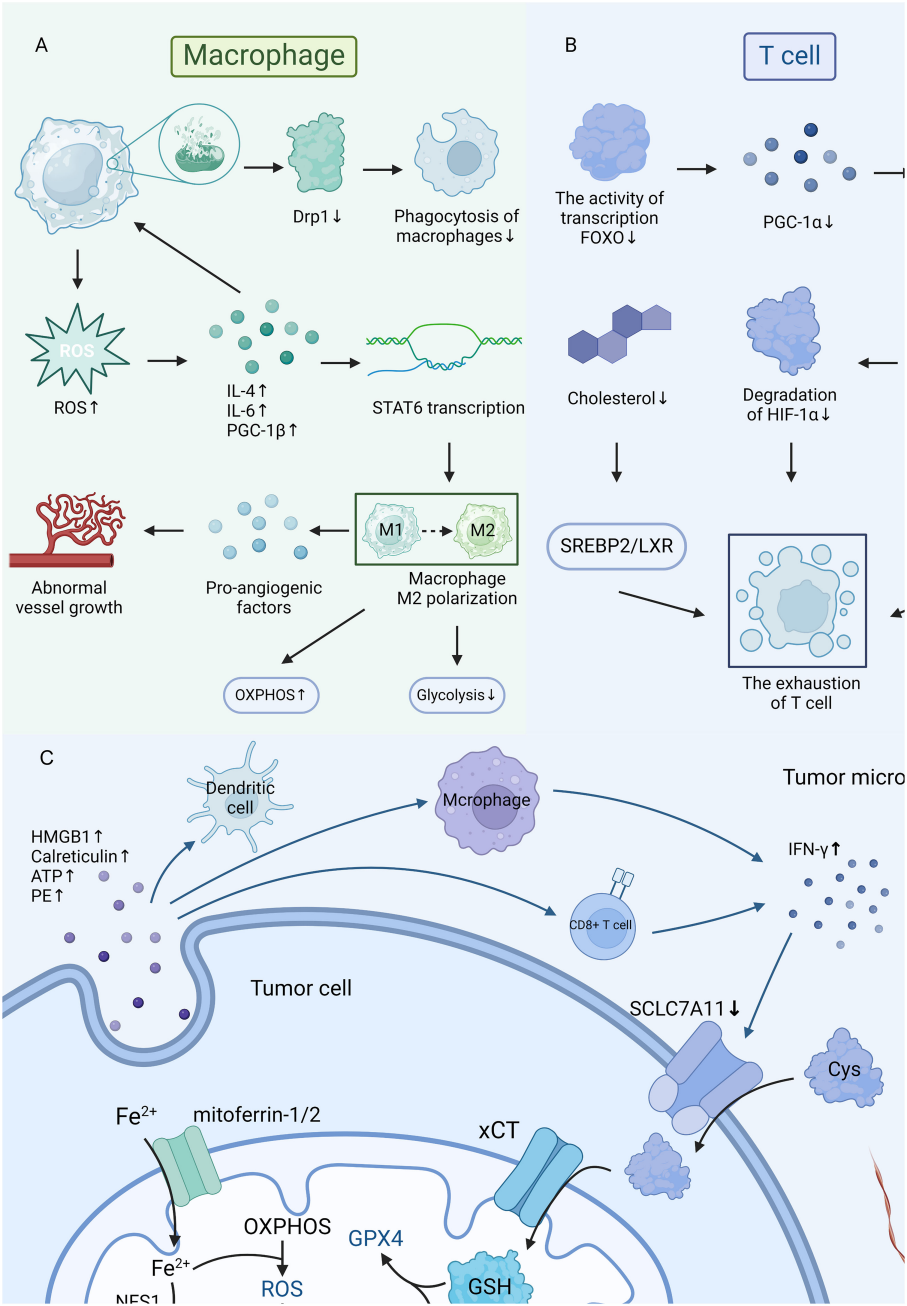
**(A, B)** The mitochondria damage in macrophages and T cells. **(C)** The connections in mitochondria, ferroptosis, and tumor microenvironment.

Mitochondrial damage in T cells is also associated with TME, especially CD8^+^ T cells and regulatory T cells (Tregs). Mitochondrial damage in T cells will result in exhaustion (44), reducing the immune responses, as shown in **Figure 2A** and **Figure 2B**. However, further researches are required to understand its implications fully. Though merely 10% of the T cells recruited in TME are thought to be capable of identifying tumor tissue, and the remaining T cells cannot eradicate tumor cells (43), T cells still key roles in immune responses against tumors (45). Notably, the activity and longevity of mitochondria are crucial in keeping the constant functions in T cells (24). Mitochondrial damage will lead to the dysfunction of mitophagy and reduce OXPHOS, which will further restrict the development and regeneration of T cells (44). In CD8^+^ T cells, researches demonstrate that the generation of excessive ROS will trigger the NFAT signal as tyrosine phosphatase inhibitors, and thus promote the transcriptional processes of CD8^+^ T depletion (46). Mitochondrial damage and cholesterol metabolism were closely connected, and it has been observed that cholesterol deficiency will result in the elimination of CD8^+^ T cells in the TME, causing the depletion and dysfunction of T cells by changing the SREBP2/LXR signaling pathway (47). Notably, CD8^+^ T cells are more sensitive to the deficiency of cholesterol than CD4^+^ T cells (47). Tregs are pivotal in maintaining immunological homeostasis and suppressing the overreaction to self-antigens (48). When mitochondria are damaged and cause the excessive generation of ROS, Tregs will be more vulnerable to oxidative stress and have a higher rate of apoptosis (49).

Currently, the research concerning the contributions of B cells with mitochondrial damage in TME is extremely limited. It is still controversial whether B cells play a supporting role or a suppressing role in tumor growth (50). However, a growing amount of research indicates that B cells are essential for anti-tumor immunity, and suggests that the presence of B lymphocytes is related to the prognosis in patients with cancer (50, 51). The research of Zhang et al. indicated that Erbin regulates mitochondrial activity in platelets, while acyl-carnitine controls mitochondrial activity and PD1 stability in B cells (52). These two factors work together to regulate the immune responses against tumors mediated by B cells (52). Additionally, the formation and the functions of the germinal center of B cells will be severely hampered by the loss of mitochondrial Translation factor A (TFAM), which is the result of mitochondrial damage, and this ultimately leads to the preventive effect of blocking the growth of tumor (53).

### 2.3 Mitochondrial damage in tumors causes ferroptosis

The cancer cell is the most essential activator of TME (10), and ferroptosis plays a key role in the growth of tumors. Ferroptosis is mainly caused by the accumulation of harmful lipid peroxides on cellular membranes (54). According to studies, mitochondrial metabolism plays an active role in ferroptosis, and ferroptotic cancer cells will exert immunology factors that affect the functions of immune cells and further alter the iron metabolism (54), as illustrated in **Figure 2C**. Interestingly, the function of mitochondria in ferroptosis was influenced by the circumstances of TME (55). Studies observed that reduced mitochondrial function can significantly suppress CDI ferroptosis, however, cells may conduct ferroptosis without the support of mitochondria if glutathione peroxidase-4 is removed or pharmacologically inhibited (55).

A high level of ROS generated by mitochondrial damage is essential in inducing ferroptosis. Studies indicated that both PINK1/Park2- and BNIP3- mediated mitochondrial autophagy have positive impacts on decreasing the generation of ROS and the expression of heme oxygenase 1, which can prevent the decrease of glutathione peroxidase 4 (GPX4) and ultimately reduces the lipid peroxidation and ferroptosis (56). Additionally, the mitochondrial glutaminolysis-TCA-ETC axis is an important pathway to ferroptosis, and it may cause ferroptosis by hastening the accumulation of lipid peroxidation (57). Ferroptosis is partially suppressed by energy stress via AMP-activated protein kinase (AMPK) and is suppressed in cancer cells with high basal AMPK activation (58). On the other side, mitochondria regulate the iron metabolism. Excessive iron may accumulate in dysfunctional mitochondria, inducing the Fenton reaction that may increase lipid peroxidation and lead to cell death (57, 59).

Depending on the composition and conditions of TME, ferroptosis plays controversial roles in carcinogenesis, restraining or promoting tumor growth (60, 61). Ferroptosis may influence the immune system to restrict the tumor growth. Research has indicated that ferroptosis may facilitate macrophage phagocytosis (61). By focusing on toll-like receptor 2 (TLR2) on macrophages, 1-steaoryl-2-15-HpETE-sn-glycero-3-phosphatidylethanolamine (SAPE-OOH) on the surface of ferroptotic cells works to transmit an eat-me signal, which directs phagocytosis and restricts the tumor growth (61). Additionally, researchers find that anti-tumor NK cells may be activated by ferroptotic cancer cells, secreting IFN-γ and causing lytic degranulation (60). However, some studies support the opposite impact, supposing ferroptosis may support the growth of tumors. Early ferroptotic tumor cells negatively impact DC antigen-presenting capacities at the transcriptional level, which can hamper the maturation of DCs, and thus inhibit adaptive immunity (62). Moreover, exogenous antigens produced by ferroptosis cells will inhibit the DC cells’ stimulation to MHC I on CD8^+^ T cells (62). Therefore, ferroptosis may lead to immunological escape by suppressing immune cells to some extent.

### 2.4 The relationship between Ca^2+^, mitochondrial damage and TME

Calcium ions, the second messenger in the cell, serve crucial parts in maintaining homeostasis and regulating signal transduction. Ca^2+^ homeostasis is necessary for a variety of cancer activities, including the growth, apoptosis, transcription of tumor cells, and vascular development of tumor tissues (63). Numerous studies have demonstrated that intracellular Ca^2+^ overloading plays a key role in inducing the enhanced generation of ROS through mitochondrial malfunction (64). High levels of ROS oxidized TRPA1 cysteine residues, upregulating Ca^2+^-dependent anti-apoptotic pathways and therefore prolong the lifespan of cancer cells (64, 65). Mitochondrial damage will result in Ca^2+^ disorder, which may lead to the overload of Ca^2+^ in cells and interfere with the conduction of signaling pathways. According to Szabadkai et al., the resistance and apoptosis sensitivity of cells are largely determined by the mitochondrial network and the interactions with endoplasmic reticulum (ER), which may affect the Ca^2+^ signal transduction (65). The feedback regulation of Ca^2+^ is affected by mitochondria, which act as intracellular Ca^2+^ buffers in close proximity to Ca^2+^ channels at the ER and the plasma membrane (66). ER-mitochondria Ca^2+^ contact sites promote the transport of Ca^2+^ into mitochondria and control IP3R channel-mediated Ca^2+^ oscillations as well as cellular metabolic processes (67).

## 3 Discussion

Mitochondria, as the powerhouses of cells, are pivotal for maintaining cellular homeostasis. However, within the TME, their function is often compromised due to hypoxia, nutrient deficiency, and heightened oxidative stress. These conditions not only compromise mitochondrial integrity but also have profound implications for the immune system and cancer progression.

Moreover, the dynamic changes of mitochondrial membrane potential and cytochrome c occupy a pivotal position in biological processes (68), Specifically, the phosphorylation status of cytochrome c significantly impacts the regulation of electron transport chain flux within mitochondria and the process of cellular apoptosis (69). Past research has confirmed that IL-22 can effectively alleviate myocardial cell apoptosis caused by reperfusion injury by blocking mitochondrial membrane potential, thereby inhibiting the production of ROS and cytochrome c (70). Recent studies have demonstrated that the prostate cancer-specific lysine 53 acetylation of cytochrome c can drive metabolic reprogramming and reduce the occurrence of apoptosis (71). Recent studies have demonstrated that the prostate cancer-specific lysine 53 acetylation of cytochrome c can drive metabolic reprogramming and reduce the occurrence of apoptosis.

It is noteworthy that mitochondrial autophagy holds tremendous promise in cancer treatment. Mitochondrial autophagy is a specific autophagic pathway in which cells eliminate damaged or no longer needed mitochondria, playing a crucial role in maintaining cellular homeostasis and responding to environmental stress (72). This process can eliminate damaged mitochondria, reduce ROS production, and suppress tumor growth (73). Activation of mitochondrial autophagy may lead to increased permeability of the mitochondrial inner membrane, releasing apoptosis-related proteins that subsequently activate cell apoptosis pathways, resulting in tumor cell death (74). Mitochondrial autophagy’ s role in maintaining cellular homeostasis and responding to environmental stress may help prevent tumor cell metastasis. However, to fully exploit the potential of mitochondrial autophagy in cancer treatment, several challenges need to be overcome. For instance, precisely regulating the process of mitochondrial autophagy to avoid adverse effects on normal cells, and integrating it with existing cancer treatment modalities, such as surgery, radiotherapy, and chemotherapy, to maximize its therapeutic effects (75). In the future, with in-depth research on mitochondrial autophagy and its regulatory mechanisms, as well as the development of new therapeutic strategies, it is believed that the potential of mitochondrial autophagy in cancer treatment will be more fully realized.

Therefore, understanding the molecular mechanisms underlying mitochondrial damage and its interaction with the immune system and TME is crucial for developing effective cancer treatments. By targeting mitochondrial dysfunction and restoring immune function, we may be able to disrupt the tumorigenic processes and achieve better therapeutic outcomes. In conclusion, this review provides a comprehensive overview of the intricate relationships between mitochondrial damage, the immune system, and the TME, offering new insights and tactics for advancing cancer treatment and immune modulation.

## Author contributions

WZ: Writing – original draft, Writing – review & editing. MW: Writing – original draft, Visualization. YZ: Writing – original draft, Visualization. TZ: Supervision, Writing – review & editing. ZZ: Funding acquisition, Supervision, Writing – review & editing.

## References

[1] Hanamura T Yokoyama K Kitano S Kagamu H Yamashita M Terao M. Investigating the immunological function of alpha-2-glycoprotein 1, zinc-binding in regulating tumor response in the breast cancer microenvironment. Cancer Immunol Immunother. (2024) 73:42. doi: 10.1007/s00262-024-03629-1 38349455 PMC10864576

[2] Bornstein R Gonzalez B Johnson SC. Mitochondrial pathways in human health and aging. Mitochondrion. (2020) 54:72–84. doi: 10.1016/j.mito.2020.07.007 32738358 PMC7508824

[3] Bow Y-D Ko C-C Chang W-T Chou S-Y Hung C-T Huang J-L. A novel quinoline derivative, DFIQ, sensitizes NSCLC cells to ferroptosis by promoting oxidative stress accompanied by autophagic dysfunction and mitochondrial damage. Cancer Cell Int. (2023) 23:171. doi: 10.1186/s12935-023-02984-w 37587444 PMC10433610

[4] Chen S Liao Z Xu P. Mitochondrial control of innate immune responses. Front Immunol. (2023) 14:1166214. doi: 10.3389/fimmu.2023.1166214 37325622 PMC10267745

[5] Xiao Y Yu D. Tumor microenvironment as a therapeutic target in cancer. Pharmacol Ther. (2021) 221:107753. doi: 10.1016/j.pharmthera.2020.107753 33259885 PMC8084948

[6] Missiroli S Perrone M Gafà R Nicoli F Bonora M Morciano G. PML at mitochondria-associated membranes governs a trimeric complex with NLRP3 and P2x7r that modulates the tumor immune microenvironment. Cell Death Differ. (2023) 30:429–41. doi: 10.1038/s41418-022-01095-9 PMC9713080 36450825

[7] Jing X Yang F Shao C Wei K Xie M Shen H. Role of hypoxia in cancer therapy by regulating the tumor microenvironment. Mol Cancer. (2019) 18:157. doi: 10.1186/s12943-019-1089-9 31711497 PMC6844052

[8] Kuo CL Ponneri Babuharisankar A Lin YC Lien HW Lo YK Chou HY. Mitochondrial oxidative stress in the tumor microenvironment and cancer immunoescape: foe or friend? J BioMed Sci. (2022) 29:74. doi: 10.1186/s12929-022-00859-2 36154922 PMC9511749

[9] Du C Guo X Qiu X Jiang W Wang X An H. Self-reinforced bimetallic mito-jammer for Ca2+ Overload-mediated cascade mitochondrial damage for cancer cuproptosis sensitization. Advanced Sci. (2024) 11:2306031. doi: 10.1002/advs.202306031 PMC11022715 38342617

[10] de Visser KE Joyce JA. The evolving tumor microenvironment: from cancer initiation to metastatic outgrowth. Cancer Cell. (2023) 41:374–403. doi: 10.1016/j.ccell.2023.02.016 36917948

[11] Quail DF Joyce JA. Microenvironmental regulation of tumor progression and metastasis. Nat Med. (2013) 19:1423–37. doi: 10.1038/nm.3394 PMC3954707 24202395

[12] Klemm F Joyce JA. Microenvironmental regulation of therapeutic response in cancer. Trends Cell Biol. (2015) 25:198–213. doi: 10.1016/j.tcb.2014.11.006 25540894 PMC5424264

[13] Bejarano L Jordāo MJC Joyce JA. Therapeutic targeting of the tumor microenvironment. Cancer Discovery. (2021) 11:933–59. doi: 10.1158/2159-8290.CD-20-1808 33811125

[14] Elia I Haigis MC. Metabolites and the tumour microenvironment: from cellular mechanisms to systemic metabolism. Nat Metab. (2021) 3:21–32. doi: 10.1038/s42255-020-00317-z 33398194 PMC8097259

[15] Zhang L Wang S Wang Y Zhao W Zhang Y Zhang N. Effects of hypoxia in intestinal tumors on immune cell behavior in the tumor microenvironment. Front Immunol. (2021) 12:645320. doi: 10.3389/fimmu.2021.645320 33737938 PMC7962475

[16] Jiang F Mao Y Lu B Zhou G Wang J. A hypoxia risk signature for the tumor immune microenvironment evaluation and prognosis prediction in acute myeloid leukemia. Sci Rep. (2021) 11:14657. doi: 10.1038/s41598-021-94128-1 34282207 PMC8289869

[17] Zhang Z Li Q Wang F Ma B Meng Y Zhang Q. Identifying hypoxia characteristics to stratify prognosis and assess the tumor immune microenvironment in renal cell carcinoma. Front Genet. (2021) 12:606816. doi: 10.3389/fgene.2021.606816 34194463 PMC8238406

[18] Suomalainen A Nunnari J. Mitochondria at the crossroads of health and disease. Cell. (2024) 187:2601–27. doi: 10.1016/j.cell.2024.04.037 38788685

[19] Bantug GR Hess C. The immunometabolic ecosystem in cancer. Nat Immunol. (2023) 24:2008–20. doi: 10.1038/s41590-023-01675-y 38012409

[20] Wu Z Xiao C Long J Huang W You F Li X. Mitochondrial dynamics and colorectal cancer biology: mechanisms and potential targets. Cell Commun Signal. (2024) 22:91. doi: 10.1186/s12964-024-01490-4 38302953 PMC10835948

[21] Li T Han J Jia L Hu X Chen L Wang Y. Pkm2 coordinates glycolysis with mitochondrial fusion and oxidative phosphorylation. Protein Cell. (2019) 10:583–94. doi: 10.1007/s13238-019-0618-z PMC6626593 30887444

[22] Chen W Zhao H Li Y. Mitochondrial dynamics in health and disease: mechanisms and potential targets. Signal Transduct Target Ther. (2023) 8:333. doi: 10.1038/s41392-023-01547-9 37669960 PMC10480456

[23] Hao S Huang H Ma RY Zeng X Duan CY. Multifaceted functions of drp1 in hypoxia/ischemia-induced mitochondrial quality imbalance: from regulatory mechanism to targeted therapeutic strategy. Mil Med Res. (2023) 10:46. doi: 10.1186/s40779-023-00482-8 37833768 PMC10571487

[24] Scharping NE Menk AV Moreci RS Whetstone RD Dadey RE Watkins SC. The tumor microenvironment represses T cell mitochondrial biogenesis to drive intratumoral T cell metabolic insufficiency and dysfunction. Immunity. (2016) 45:374–88. doi: 10.1016/j.immuni.2016.07.009 PMC5207350 27496732

[25] Liu Y Xu R Gu H Zhang E Qu J Cao W. Metabolic reprogramming in macrophage responses. biomark Res. (2021) 9:1. doi: 10.1186/s40364-020-00251-y 33407885 PMC7786975

[26] Guo W Li Z Huang H Xu Z Chen Z Shen G. VB12-Sericin-PBLG-IR780 nanomicelles for programming cell pyroptosis *via* photothermal (PTT)/photodynamic (PDT) effect-induced mitochondrial DNA (Mitodna) oxidative damage. ACS Appl Materials Interfaces. (2022) 14:17008–21. doi: 10.1021/acsami.1c22804 35394753

[27] Huang C Lin B Chen C Wang H Lin X Liu J. Synergistic reinforcing of immunogenic cell death and transforming tumor-associated macrophages *via* a multifunctional cascade bioreactor for optimizing cancer immunotherapy. Advanced Materials. (2022) 34:2207593. doi: 10.1002/adma.202207593 36245299

[28] Italiani P Boraschi D. From monocytes to M1/M2 macrophages: phenotypical vs. Functional differentiation. Front Immunol. (2014) 5:514. doi: 10.3389/fimmu.2014.00514 25368618 PMC4201108

[29] Liu Q Yan X Li R Yuan Y Wang J Zhao Y. Polyamine signal through hcc microenvironment: A key regulator of mitochondrial preservation and turnover in tams. Int J Mol Sci. (2024) 25:996. doi: 10.3390/ijms25020996 38256070 PMC10816144

[30] Yurdagul A. Metabolic consequences of efferocytosis and its impact on atherosclerosis. Immunometabolism. (2021) 3:e210017. doi: 10.20900/immunometab20210017 33927896 PMC8081385

[31] Batista-Gonzalez A Vidal R Criollo A Carreño LJ. New insights on the role of lipid metabolism in the metabolic reprogramming of macrophages. Front Immunol. (2020) 10:2993. doi: 10.3389/fimmu.2019.02993 31998297 PMC6966486

[32] Kim S-H Kim M-J Lim M Kim J Kim H Yun C-K. Enhancement of the anticancer ability of natural killer cells through allogeneic mitochondrial transfer. Cancers. (2023) 15:3225. doi: 10.3390/cancers15123225 37370835 PMC10296914

[33] Weber M Ries J Braun K Wehrhan F Distel L Geppert C. Neoadjuvant radiochemotherapy alters the immune and metabolic microenvironment in oral cancer-analyses of CD68, CD163, TGF-Β1, GLUT-1 and HIF-1α Expressions. Cells. (2024) 13:397. doi: 10.3390/cells13050397 PMC10930773 38474362

[34] Li Q Chen C Kong J Li L Li J Huang Y. Stimuli-responsive nano vehicle enhances cancer immunotherapy by coordinating mitochondria-targeted immunogenic cell death and Pd-L1 blockade. Acta Pharm Sin B. (2022) 12:2533–49. doi: 10.1016/j.apsb.2021.11.005 PMC9136536 35646521

[35] Brand MD Nicholls DG. Assessing mitochondrial dysfunction in cells. Biochem J. (2011) 435:297–312. doi: 10.1042/BJ20110162 21726199 PMC3076726

[36] Li Y Liu J Chen Y Weichselbaum RR Lin W. Nanoparticles synergize ferroptosis and cuproptosis to potentiate cancer immunotherapy. Adv Sci (Weinh). (2024) 11:e2310309. doi: 10.1002/advs.202310309 38477411 PMC11187894

[37] Liang T Feng Z Zhang X Li T Yang T Yu L. Research progress of calcium carbonate nanomaterials in cancer therapy: challenge and opportunity. Front Bioengineering Biotechnol. (2023) 11:1266888. doi: 10.3389/fbioe.2023.1266888 PMC10551635 37811375

[38] Nejadi Orang F Abdoli Shadbad M. Competing endogenous RNA networks and ferroptosis in cancer: novel therapeutic targets. Cell Death Dis. (2024) 15:357. doi: 10.1038/s41419-024-06732-4 38778030 PMC11111666

[39] Liu S Duan C Xie J Zhang J Luo X Wang Q. Peripheral immune cell death in sepsis based on bulk RNA and single-cell RNA sequencing. Heliyon. (2023) 9:e17764. doi: 10.1016/j.heliyon.2023.e17764 37455967 PMC10339024

[40] Zimmerman O Olbrich P Freeman AF Rosen LB Uzel G Zerbe CS. Stat1 gain-of-function mutations cause high total stat1 levels with normal dephosphorylation. Front Immunol. (2019) 10:1433. doi: 10.3389/fimmu.2019.01433 31354696 PMC6635460

[41] Xu B Tang B Wei J. Role of STAT1 in the resistance of HBV to IFN−Α. Exp Ther Med. (2021) 21:1–8. doi: 10.3892/etm.2021.9982 33850522 PMC8027746

[42] Butturini E Carcereri de Prati A Mariotto S. Redox regulation of STAT1 and STAT3 signaling. Int J Mol Sci. (2020) 21:7034. doi: 10.3390/ijms21197034 32987855 PMC7582491

[43] Qian Y Chen W Wang M Xie Y Qiao L Sun Q. Tumor microenvironment-specific driven nanoagents for synergistic mitochondria damage-related immunogenic cell death and alleviated immunosuppression. Small Methods. (2023):e2301231. doi: 10.1002/smtd.202301231 38126694

[44] Li W Cheng H Li G Zhang L. Mitochondrial damage and the road to exhaustion. Cell Metab. (2020) 32:905–7. doi: 10.1016/j.cmet.2020.11.004 33264601

[45] Zhang L Romero P. Metabolic control of CD8(+) T cell fate decisions and antitumor immunity. Trends Mol Med. (2018) 24:30–48. doi: 10.1016/j.molmed.2017.11.005 29246759

[46] Schieber M Chandel NS. Ros function in redox signaling and oxidative stress. Curr Biol. (2014) 24:R453–62. doi: 10.1016/j.cub.2014.03.034 PMC4055301 24845678

[47] Yan C Zheng L Jiang S Yang H Guo J Jiang LY. Exhaustion-associated cholesterol deficiency dampens the cytotoxic arm of antitumor immunity. Cancer Cell. (2023) 41:1276–93.e11. doi: 10.1016/j.ccell.2023.04.016 37244259

[48] Yan Y Huang L Liu Y Yi M Chu Q Jiao D. Metabolic profiles of regulatory T cells and their adaptations to the tumor microenvironment: implications for antitumor immunity. J Hematol Oncol. (2022) 15:104. doi: 10.1186/s13045-022-01322-3 35948909 PMC9364625

[49] Maj T Wang W Crespo J Zhang H Wang W Wei S. Oxidative stress controls regulatory T cell apoptosis and suppressor activity and PD-L1-blockade resistance in tumor. Nat Immunol. (2017) 18:1332–41. doi: 10.1038/ni.3868 PMC5770150 29083399

[50] Downs-Canner SM Meier J Vincent BG Serody JS. B cell function in the tumor microenvironment. Annu Rev Immunol. (2022) 40:169–93. doi: 10.1146/annurev-immunol-101220-015603 35044794

[51] Sharonov GV Serebrovskaya EO Yuzhakova DV Britanova OV Chudakov DM. B cells, plasma cells and antibody repertoires in the tumour microenvironment. Nat Rev Immunol. (2020) 20:294–307. doi: 10.1038/s41577-019-0257-x 31988391

[52] Zhang Z Xu X Zhang D Zhao S Wang C Zhang G. Targeting Erbin-mitochondria axis in platelets/megakaryocytes promotes B cell-mediated antitumor immunity. Cell Metab. (2024) 36:541–56.e9. doi: 10.1016/j.cmet.2023.12.020 38232736

[53] Yazicioglu YF Marin E Sandhu C Galiani S Raza IGA Ali M. Dynamic mitochondrial transcription and translation in B cells control germinal center entry and lymphomagenesis. Nat Immunol. (2023) 24:991–1006. doi: 10.1038/s41590-023-01484-3 37095377 PMC10232359

[54] Lei G Zhuang L Gan B. Targeting ferroptosis as a vulnerability in cancer. Nat Rev Cancer. (2022) 22:381–96. doi: 10.1038/s41568-022-00459-0 PMC10243716 35338310

[55] Gao M Yi J Zhu J Minikes AM Monian P Thompson CB. Role of mitochondria in ferroptosis. Mol Cell. (2019) 73:354–63.e3. doi: 10.1016/j.molcel.2018.10.042 30581146 PMC6338496

[56] Lin Q Li S Jin H Cai H Zhu X Yang Y. Mitophagy alleviates cisplatin-induced renal tubular epithelial cell ferroptosis through ROS/HO-1/GPX4 axis. Int J Biol Sci. (2023) 19:1192–210. doi: 10.7150/ijbs.80775 PMC10008689 36923942

[57] Cheng X Zhang J Xiao Y Wang Z He J Ke M. Mitochondrial regulation of ferroptosis in cancer therapy. Int J Mol Sci. (2023) 24:10037. doi: 10.3390/ijms241210037 PMC10298270 37373183

[58] Lee H Zandkarimi F Zhang Y Meena JK Kim J Zhuang L. Energy-stress-mediated AMPK activation inhibits ferroptosis. Nat Cell Biol. (2020) 22:225–34. doi: 10.1038/s41556-020-0461-8 PMC7008777 32029897

[59] Kajarabille N Latunde-Dada GO. Programmed cell-death by ferroptosis: antioxidants as mitigators. Int J Mol Sci. (2019) 20:4968. doi: 10.3390/ijms20194968 PMC6801403 31597407

[60] Sang R Fan R Deng A Gou J Lin R Zhao T. Degradation of hexokinase 2 blocks glycolysis and induces GSDME-dependent pyroptosis to amplify immunogenic cell death for breast cancer therapy. J Medicinal Chem. (2023) 66:8464–83. doi: 10.1021/acs.jmedchem.3c00118 37376788

[61] Luo X Gong H-B Gao H-Y Wu Y-P Sun W-Y Li Z-Q. Oxygenated phosphatidylethanolamine navigates phagocytosis of ferroptotic cells by interacting with TLR2. Cell Death Differentiation. (2021) 28:1971–89. doi: 10.1038/s41418-020-00719-2 PMC8185102 33432112

[62] Wiernicki B Maschalidi S Pinney J Adjemian S Vanden Berghe T Ravichandran KS. Cancer cells dying from ferroptosis impede dendritic cell-mediated anti-tumor immunity. Nat Commun. (2022) 13:3676. doi: 10.1038/s41467-022-31218-2 35760796 PMC9237053

[63] Song Y Yang Z Wang X Ye Y Yan X Huang Y. Harnessing a triphenylphosphine-based AIE nano-platform for triggering incomplete mitophagy to continuously augment anti-tumor immune response in hepatocellular carcinoma. Nano Today. (2024) 54:102090. doi: 10.1016/j.nantod.2023.102090

[64] Wang SZ Guo Y Zhang X Feng HH Wu SY Zhu YX. Mitochondria-targeted photodynamic and mild-temperature photothermal therapy for realizing enhanced immunogenic cancer cell death *via* mitochondrial stress. Advanced Funct Materials. (2023) 33:2303328. doi: 10.1002/adfm.202303328

[65] Shandilya S Ruokolainen J. Modulating effects of vitamin K2 on oxidative stress induced organelle damage in alzheimer’s disease. Alzheimer's Dementia. (2023) 19:e078731. doi: 10.1002/alz.078731

[66] Rizzuto R De Stefani D Raffaello A Mammucari C. Mitochondria as sensors and regulators of calcium signalling. Nat Rev Mol Cell Biol. (2012) 13:566–78. doi: 10.1038/nrm3412 22850819

[67] Booth DM Enyedi B Geiszt M Várnai P Hajnóczky G. Redox nanodomains are induced by and control calcium signaling at the ER-mitochondrial interface. Mol Cell. (2016) 63:240–8. doi: 10.1016/j.molcel.2016.05.040 PMC4998968 27397688

[68] Brazhe NA Nikelshparg EI Baizhumanov AA Grivennikova VG Semenova AA Novikov SM. Sers uncovers the link between conformation of cytochrome C heme and mitochondrial membrane potential. Free Radic Biol Med. (2023) 196:133–44. doi: 10.1016/j.freeradbiomed.2023.01.013 36649901

[69] Kalpage HA Wan J Morse PT Zurek MP Turner AA Khobeir A. Cytochrome C phosphorylation: control of mitochondrial electron transport chain flux and apoptosis. Int J Biochem Cell Biol. (2020) 121:105704. doi: 10.1016/j.biocel.2020.105704 32023432 PMC7044036

[70] Che Y Tian Y Chen R Xia L Liu F Su Z. IL-22 ameliorated cardiomyocyte apoptosis in cardiac ischemia/reperfusion injury by blocking mitochondrial membrane potential decrease, inhibiting ROS and cytochrome C. Biochim Biophys Acta Mol Basis Dis. (2021) 1867:166171. doi: 10.1016/j.bbadis.2021.166171 34015450

[71] Morse PT Wan J Arroum T Herroon MK Kalpage HA Bazylianska V. Prostate cancer-specific lysine 53 acetylation of cytochrome C drives metabolic reprogramming and protects from apoptosis in intact cells. Biomolecules. (2024) 14:695. doi: 10.3390/biom14060695 38927098 PMC11201891

[72] Payen VL Zampieri LX Porporato PE Sonveaux P. Pro- and antitumor effects of mitochondrial reactive oxygen species. Cancer Metastasis Rev. (2019) 38:189–203. doi: 10.1007/s10555-019-09789-2 30820778

[73] Wang T He M Zhang X Guo Z Wang P Long F. Deciphering the impact of circrna-mediated autophagy on tumor therapeutic resistance: A novel perspective. Cell Mol Biol Lett. (2024) 29:60. doi: 10.1186/s11658-024-00571-z 38671354 PMC11046940

[74] Reyes-Castellanos G Abdel Hadi N Carrier A. Autophagy contributes to metabolic reprogramming and therapeutic resistance in pancreatic tumors. Cells. (2022) 11:426. doi: 10.3390/cells11030426 35159234 PMC8834004

[75] Kopecka J Gazzano E Castella B Salaroglio IC Mungo E Massaia M. Mitochondrial metabolism: inducer or therapeutic target in tumor immune-resistance? Semin Cell Dev Biol. (2020) 98:80–9. doi: 10.1016/j.semcdb.2019.05.008 31100351

