# Corrigendum: Targeting mitochondrial damage: shining a new light on immunotherapy

**DOI:** 10.3389/fimmu.2025.1591209

**Published:** 2025-03-20

**Authors:** Wenjuan Zeng, Menghui Wang, Yuxin Zhang, Taicheng Zhou, Zhen Zong

**Affiliations:** ^1^ Department of Gastrointestinal Surgery, The 2Affiliated Hospital, Jiangxi Medical College, Nanchang University, Nanchang, Jiangxi, China; ^2^ Huan Kui Academy, Jiangxi Medical College, Nanchang University, Nanchang, Jiangxi, China; ^3^ The Second Clinical Medical College, Jiangxi Medical College, Nanchang University, Nanchang, Jiangxi, China; ^4^ Department of Gastroenterological Surgery and Hernia Center, The Sixth Affiliated Hospital, Sun Yat-sen University, Guangdong Provincial Key Laboratory of Colorectal and Pelvic Floor Diseases, Guangzhou, China

**Keywords:** mitochondrial damage, immunotherapy, tumor microenvironment, target, immune cells

In the published article, there was an error in **Figure 1** as published. We have created a new **Figure 1** and this new Figure provides a more detailed description of the mechanisms. The corrected **Figure 1** and its caption: “The main mechanisms and characteristics of mitochondrial damage” appear below.

**Figure 1:**
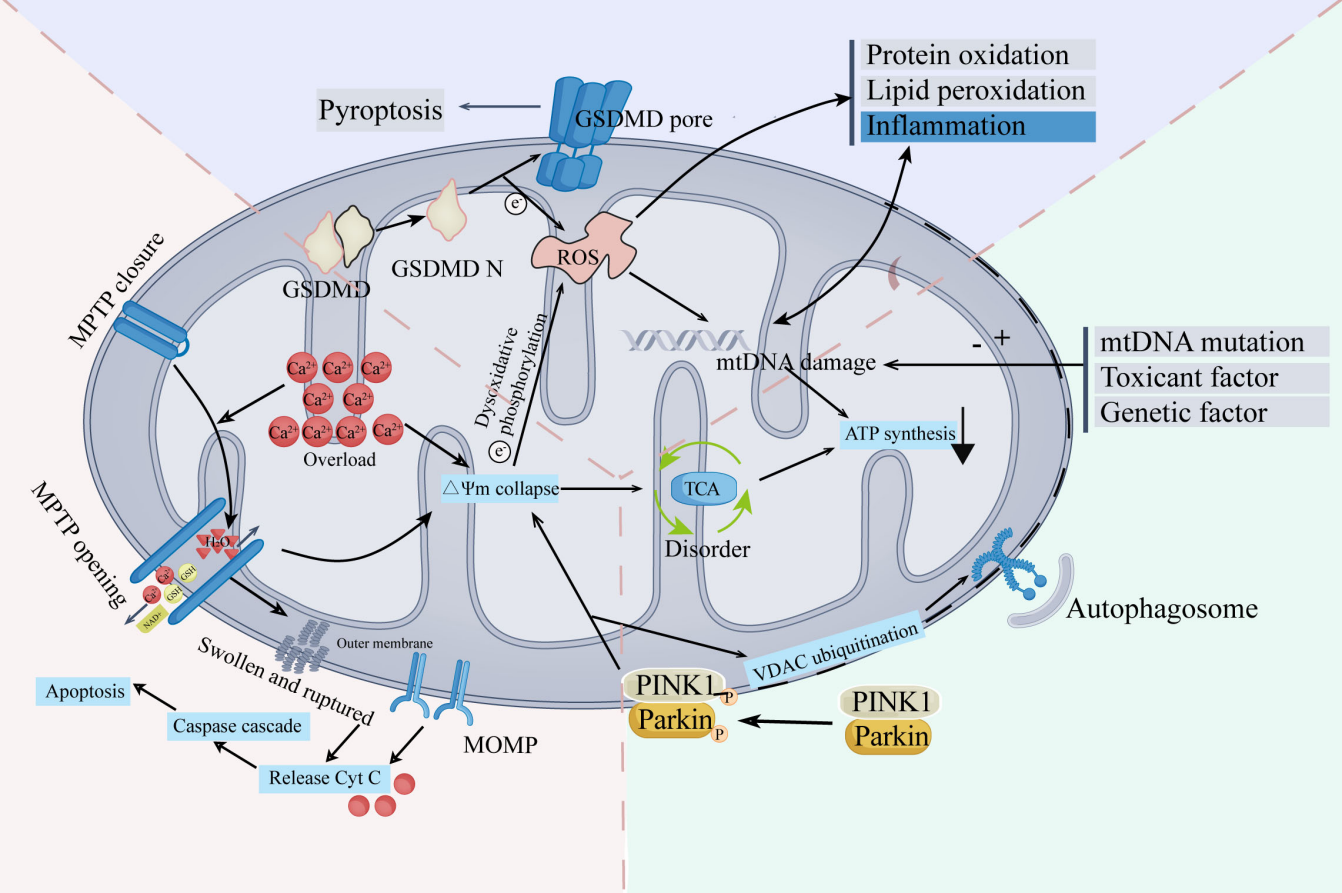
The main mechanisms and characteristics of mitochondrial damage.

The authors apologize for this error and state that this does not change the scientific conclusions of the article in any way. The original article has been updated.

